# TriQuery-BEV: Enhancing 3D Perception for Autonomous Driving with Temporal Query Filtering and Uncertainty-Aware Fusion

**DOI:** 10.3390/s26102934

**Published:** 2026-05-07

**Authors:** Junyi Dong, Xuemei Chen, Zemin Liu

**Affiliations:** School of Mechanical Engineering, Beijing Institute of Technology, 5 South Zhongguancun Street, Haidian District, Beijing 100811, China; 3220240489@bit.edu.cn (J.D.); 3220240492@bit.edu.cn (Z.L.)

**Keywords:** 3D perception, bird’s-eye view perception, temporal query fusion

## Abstract

Existing BEV perception methods unify multi-view information in a bird’s-eye-view coordinate system, yet their performance in dynamic traffic scenes remains limited by three major error sources: depth-noise amplification during image-to-BEV lifting, representation discontinuity caused by time-varying occlusion and visibility, and temporal drift induced by recursive fusion of historical BEV features. To address these issues while preserving computational tractability, we propose TriQuery-BEV, a modular enhancement framework over BEVFormer that improves BEV query modeling from the perspectives of geometric ambiguity, occlusion robustness, and temporal consistency. The proposed framework integrates three components: Query Mask (QM) for structured regularization in the BEV query space, depth-modulated hybrid positional encoding (DM-HPE) for geometry-aware positional representation, and a Temporal Query Filter (TQF) for uncertainty-aware temporal fusion. Experiments on the nuScenes benchmark demonstrate consistent improvements over BEVFormer across different model scales. TriQuery-BEV improves the nuScenes detection score (NDS) and mean average precision (mAP) by 5.4%/6.4% under the Tiny (ResNet-50) setting and by 6.0%/6.5% under the Base (ResNet-101) setting. It also reduces key true-positive error metrics, including mean translation error (mATE) by 2.9%/6.5%, mean orientation error (mAOE) by 5.7%/8.3%, and mean velocity error (mAVE) by 7.3%/15.0% for Tiny/Base, respectively. Extensive ablations further verify the effectiveness of DM-HPE, TQF, and QM, confirming improved robustness, geometric accuracy, and temporal consistency in highly dynamic environments.

## 1. Introduction

### 1.1. Motivation and Overview

The bird’s-eye view (BEV) representation is a key paradigm for 3D environmental understanding in autonomous driving, due to its natural coupling with vehicle kinematics and ease of cross-sensor fusion. BEVFormer [1], a purely visual approach, aggregates multi-view image features into BEV queries via temporal self-attention and spatial cross-attention, achieving competitive performance in 3D detection and BEV perception tasks on platforms like nuScenes. However, the “image-to-BEV” end-to-end mapping faces three core challenges: (1) dynamic object motion causes accumulated errors in localization and motion estimation, thereby weakening temporal consistency across frames; (2) occlusions and image degradation, leading to feature loss and instability in BEV query updates; and (3) drift in cross-frame fusion, which weakens temporal consistency. In environments such as logistics parks and mining areas, dust, fog, and reflective surfaces further complicate feature discriminability, increasing erroneous associations. Moreover, real-time constraints prevent the indefinite expansion of historical frames or resolution, requiring a balance between speed and accuracy.

To address these challenges, we propose TriQuery-BEV, a BEVFormer-based modular enhancement framework that revisits BEV query updates under three practical failure modes: depth-noise amplification, occlusion-induced discontinuity, and temporal drift. We introduce Query Mask (QM), which applies structured dropout to the BEV query space during training to improve robustness under partial query corruption, while being bypassed at inference. To tackle geometric uncertainty, we combine random Fourier features with learnable encodings, and apply Feature-wise Linear Modulation (FiLM) [2] for adaptive calibration based on depth, improving accuracy in ambiguous areas. To mitigate temporal drift, we design Temporal Query Filter (TQF), estimating and fusing uncertainty in predictions and observations for more consistent temporal fusion. TriQuery-BEV is designed to address these challenges and shows consistent improvements in dynamic driving scenes.

The main contributions of this paper can be summarized in three points:

1. We formulate three practical query-level failure modes in camera-only BEV perception within the BEVFormer pipeline: depth-noise amplification, occlusion-induced representation discontinuity, and temporal drift from recursive fusion.
2. We present a modular enhancement strategy that addresses these issues through training-time query regularization (QM), depth-modulated hybrid positional modulation (DM-HPE), and uncertainty-guided temporal refinement (TQF).
3. We demonstrate consistent improvements across both Tiny and Base variants on nuScenes, with gains in NDS/mAP and reductions in key error metrics, indicating that the proposed integration improves geometric stability and temporal consistency in a practically meaningful manner.

We are organizing all the code and will be releasing it as open-source in the future.

### 1.2. Related Works

#### 1.2.1. BEV-Based 3D Perception Frameworks

The bird’s-eye view (BEV) representation has become a universal perception model for 3D perception tasks in autonomous driving due to its natural coupling with vehicle kinematics and its ease of multi-sensor alignment. Early methods such as LSS (Lift, Splat, Shoot) [3] explicitly lifted 2D features into 3D space using depth estimation. However, due to high computational costs, they were not suitable for real-time applications. Building on this, BEVFormer introduced the groundbreaking spatiotemporal cross-attention mechanism, directly aggregating multi-view images into BEV queries, significantly enhancing performance in 3D object detection and semantic understanding tasks, particularly achieving state-of-the-art results on the nuScenes dataset [4]. Subsequent research has primarily focused on optimizing model efficiency. BEVFormerV2 incorporated a perspective supervision mechanism, using 2D detection and depth estimation tasks as auxiliary branches to force BEV features to retain fine-grained geometric information, thus accelerating the convergence of the image backbone network and improving stability in dynamic scenes [5]. SparseBEV generated sparse 3D candidate points from images and used sparse convolution to process BEV features, avoiding dense computation in empty regions [6]. RecurrentBEV introduced efficient temporal fusion strategies that optimized memory consumption, making real-time deployment on large-scale datasets possible [7]. Although these methods perform well in closed-vocabulary detection tasks, their visual representations are still limited by pure image supervision and struggle to generalize to open-vocabulary scenarios. To address this issue, CLIP-BEVFormer explored distilling CLIP’s vision–language pretraining knowledge into BEV queries and generating open semantic priors through text prompts, significantly improving detection capabilities for unknown categories [8]. However, such methods rely on large-scale textual annotations and introduce additional computational burdens, affecting inference efficiency. Recent multimodal visual analysis has also shown that combining spatial-frequency cues and motion cues can improve robustness under visually challenging conditions, which is broadly consistent with our motivation for strengthening BEV perception under degraded environments [9]. In this work, we enhance the representation by explicitly modulating geometry and modeling uncertainty, all while maintaining pure visual input.

#### 1.2.2. Positional Encoding with Geometric Priors

Positional encoding is crucial for 3D spatial reasoning. BEVFormer employs learnable positional encodings, which provide flexibility but can lead to frame inconsistency in depth-ambiguous regions. Recent works have attempted to inject explicit geometric priors: PETR [10] uses 3D coordinate encoding, and Sparse4D [11,12,13] introduces intrinsic and extrinsic projection encodings. Furthermore, RoPETR [14], based on StreamPETR [15], incorporates enhanced rotational positional encoding (Rotary Position Embedding), injecting spatial and temporal positional information into query representations in a composable rotational form, thus strengthening temporal motion modeling and improving speed estimation performance.

Beyond the BEV detection setting, the value of explicitly injecting camera geometry into positional representations has also been recognized in broader multi-view 3D analysis. For example, GMViT [16] introduces spatial encoding of camera/view coordinates as position embeddings in a hierarchical multi-view transformer for 3D shape analysis. More specifically, it maps camera rendering coordinates to learned positional embeddings at the view level and further propagates grouped spatial coordinates to the group-level transformer. This line of work is relevant to our discussion because it highlights the importance of geometry-aware positional representation. However, it is designed for static multi-view 3D shape analysis rather than query-based BEV perception in dynamic driving scenes. MonoDETR [17] implicitly models and explicitly utilizes scene geometry by converting the predicted foreground depth map into global depth context and guiding object queries to adaptively aggregate image features in end-to-end monocular 3D detection. Random Fourier Features (RFF) have been theoretically proven to be robust to distribution shifts [18], but have yet to be applied in the BEV perception domain. In our method, we combine RFF with learnable encodings and apply reliability-guided Feature-wise Linear Modulation (FiLM) through a lightweight gating branch, thereby improving geometric consistency in depth-ambiguous regions while retaining the adaptability of learnable positional representations.

#### 1.2.3. Temporal Fusion via Uncertainty Estimation

Temporal fusion is a core aspect of dynamic scene understanding. Existing BEV perception methods often implicitly aggregate historical representations through attention mechanisms or memory units, but few explicitly model the state uncertainty during temporal updates, leading to representation drift under high dynamics or long temporal sequences with accumulated errors. Recent works have introduced probabilistic modeling into 3D perception: MonoDETR employs distribution parameterization in 3D bounding box regression to estimate prediction uncertainty; Monte Carlo Dropout [19] has also been used in ST-P3 [20] for confidence evaluation in end-to-end planning. However, such probabilistic methods often introduce additional inference overhead due to sampling or instability during training. UAP-BEV [21] attempts to incorporate uncertainty modeling into downstream planning tasks, achieving probabilistic collision avoidance through sampling optimization. However, it focuses on decision uncertainty at the planning layer rather than feature update uncertainty at the perception layer. In contrast, Kalman filtering has been extensively validated in multi-source BEV detection fusion and multi-object tracking [22,23], but applying its principles to BEV feature-level temporal updates presents several challenges. Key among these are: constructing a lightweight prediction–observation model compatible with attention mechanisms, estimating observation uncertainty using internal attention statistics, and avoiding full covariance matrix calculations to ensure efficient parallel updates for large-scale queries.

## 2. Materials and Methods

### 2.1. Overall Architecture

**Figure 1:**
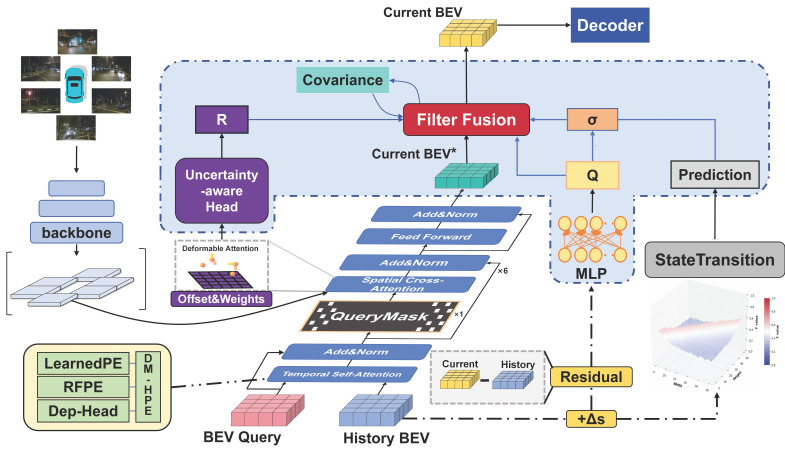
The overall architecture of TriQuery-BEV is shown. The asterisk (*) represents the unfiltered BEV features from the baseline encoder, which are not involved in the residual calculation of the Q branch. The heatmap of the Prediction branch visualizes the single-layer state transition matrix, with its amplitude reduced for easier visualization. σ is the regulator to prevent the Prediction from dominating the filtering process and diluting the encoder’s output.

TriQuery-BEV is built on the BEVFormer encoder–decoder framework. It introduces three mechanisms that target the geometric modeling, occlusion robustness, and temporal consistency of BEV queries, thereby improving perception under dynamic, occluded, and geometrically complex conditions. The overall framework is illustrated in Figure 1. Given a multi-view image feature sequence $F_{t}\in R^{B\times C\times N\times H\times W}(t=1,2,\ldots ,T)$, the network first uses a lightweight depth-reliability head to generate the gating map D_t_, which helps the model better handle depth uncertainty through hybrid positional modulation. Then, the encoder receives the current BEV query $Q\_t\in R^{Nq\times B\times C}$ and the previous query Q_t−1_, performing temporal self-attention (TSA), Query Mask (QM), and spatial cross-attention (SCA) sequentially to output the unfiltered feature Z_t_. Next, the uncertainty extraction head collects attention weights and sampling offsets from the SCA module, derives diagonal variance cues for each query, and applies a Kalman-style update to obtain the corrected query. Here, the learned variance is used as a feature-level reliability signal for temporal refinement, rather than as a fully calibrated probabilistic uncertainty estimate. Finally, the detection decoder uses the corrected query Q_t_ as input and outputs 3D bounding boxes and semantic categories.

During feature extraction, the network employs ResNet as the backbone, combined with a Feature Pyramid Network (FPN) for multi-scale feature extraction. The generated 2D feature tensor, with shape B × N_cam_ × C × H × W, is further processed to produce the depth-reliability gating map D_t_, which is then used in the hybrid positional encoding module. In the encoder part, the BEV query and historical queries are processed by multiple stacked encoding blocks, where the temporal self-attention (TSA) module effectively models temporal dependencies, enhancing the query’s reliance on historical information. The Query Mask (QM) module randomly drops values from the BEV query space, forcing the model to learn how to handle local occlusions and information loss during training. The spatial cross-attention (SCA) module aggregates cross-view features, improving the spatial semantic representation capability.

To improve temporal consistency in dynamic environments, TriQuery-BEV introduces the Temporal Query Filter (TQF), which performs query-level temporal refinement using learned diagonal variance cues derived from the current observation and temporally propagated state. This design helps reduce query drift in highly dynamic scenes and improves cross-frame consistency. The decoder portion of the model performs multi-level feature fusion and incremental query representation optimization, ultimately outputting multi-resolution semantic occupancy grids and 3D bounding boxes. During inference, TriQuery-BEV disables only the Query Mask (QM) module, since it serves as a training-time regularizer. By contrast, the Temporal Query Filter (TQF) remains enabled during inference and performs query-level temporal refinement in the reported evaluation results. The depth reliability gating map in DM-HPE is also retained during inference as part of the geometry-aware positional modulation process. Notably, TQF reuses internal attention statistics and adopts diagonal variance updates, which keeps the additional computation limited in the current implementation. Through these plug-and-play modules, TriQuery-BEV improves perception quality in dynamic environments while preserving architectural simplicity.

### 2.2. Depth-Modulated Hybrid Positional Encoding

**Figure 2:**
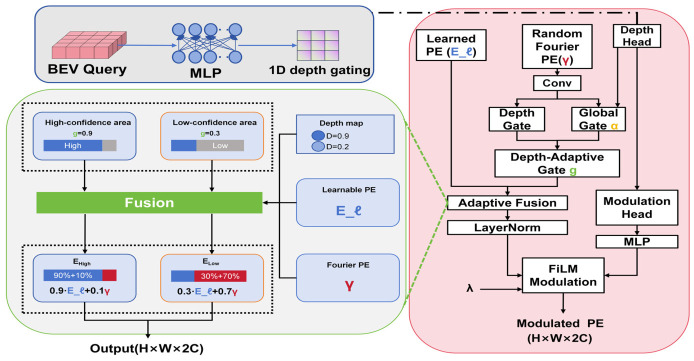
Single-layer depth-adaptive gating mechanism and fused positional encoding.

This section introduces Depth-Modulated Hybrid Positional Encoding (DM-HPE). As shown in Figure 2, DM-HPE establishes a unified positional encoding mechanism for BEV queries by coupling spectral-domain geometric priors with depth-reliability modulation, thereby improving geometric alignment and cross-scene generalization ability. BEVFormer uses purely learnable positional encodings $E_{l}\in R^{H\times W\times C}$ to provide positional information for BEV queries. While this approach offers a high degree of representational flexibility, it lacks explicit geometric prior constraints, leading to inconsistencies in cross-frame representation, especially in depth-ambiguous regions (such as elevated and ground overlapping areas). To inject extrapolable geometric priors while maintaining data-driven capabilities, this study proposes Depth-Modulated Hybrid Positional Encoding (DM-HPE), which replaces the original Learned Positional Encoding (PE) as the positional encoding term for BEV queries. The process is shown in Figure 1: (1) Spectral domain priors are generated using Random Fourier Features (RFF). (2) The depth reliability gating map is predicted. (3) The learnable encoding is fused with the predicted depth reliability map to form a hybrid positional representation, and Feature-wise Linear Modulation (FiLM) is applied, utilizing the depth reliability gating map to adaptively modulate the hybrid encoding. This achieves a closed-loop correction between “depth ambiguity” and “positional encoding”.

It should be emphasized that the depth branch in DM-HPE is not designed to predict metrically accurate depth. Instead, it provides a learned local reliability cue used to modulate the hybrid positional encoding in depth-ambiguous regions.

The Random Fourier Features (RFF) spectral domain prior is computed based on the normalized BEV grid coordinates p = (x, y) ∈ [−1, 1]^2^, using Random Fourier Mapping

$$\gamma \left(p\right)=\left[\cos\left(2\pi \mathrm{Bp}\right);\sin\left(2\pi \mathrm{Bp}\right)\right],B\in R^{m\times 2}.$$

The elements of B are sampled from a normal distribution $N\left(0,\sigma^{2}\right)$, where σ controls the wavelength distribution. B is sampled once during initialization and kept fixed thereafter. This mapping transforms the 2D coordinates into a 2m-dimensional differentiable spectral feature, providing the network with an explicit spectral basis and distance-sensitive priors for subsequent processing. The depth reliability gating is estimated to perform adaptive modulation in depth-ambiguous regions. The model predicts a per-location reliability gate for the BEV query features $Q_{\mathrm{bev}}\in R^{H\times W\times C}$, allowing for adaptive modulation based on the depth ambiguity, which helps improve the robustness of the model in regions where depth information is unclear.

$$d=\sigma \left({\mathrm{Conv}}_{1\times 1}^{\left(2\right)}\left(\phi \left({\mathrm{Conv}}_{1\times 1}^{\left(1\right)}\left(Q_{\mathrm{bev}}\right)\right)\right)\right)$$

Here, ϕ(·) represents a lightweight nonlinear activation function (ReLU), and σ(·) represents the Sigmoid function. The two-layer 1 × 1 Bottleneck head (C→r→1) has a parameter count of approximately 8.3 k in a typical setup (e.g., C = 256, r = 32), indicating that the added parameter cost is small. It is important to note that d is used to describe local depth reliability and serves as a gating signal for positional encoding modulation, rather than being explicitly used as a depth regression quantity. Combined with the learnable global gate α, this forms a per-pixel gating mechanism:

g=Clamp(σ(α)+d−0.5)∈[0.05,0.95]

Here, α∈R^{2C}^ represents the learnable parameter. The final hybrid encoding is obtained through gating-based weighted fusion as follows:

$$E_{h}=g⊙E_{l}+\left(1-g\right)⊙\gamma$$

This design adjusts the contribution of the two encodings according to depth reliability. In geometrically reliable regions, the model relies more on the learnable encoding. In depth-ambiguous regions, it relies more on the Fourier basis to reduce positional drift. To further refine geometry-aware positional encoding, FiLM is applied to adaptively perform affine correction on the hybrid encoding. The model generates per-pixel modulation parameters from the depth reliability gating map using a lightweight Multi-Layer Perceptron (MLP):

$$\left(\gamma_{film},\beta_{film}\right)=MLP\left(D\right)\in R^{H\times W\times 2C}$$

The MLP consists of two 1 × 1 convolution layers. The final output is obtained through a residual connection, as follows:

$$E_{out}=E_{h}+\lambda \cdot \left(\gamma_{film}⊙LN\left(E_{h}\right)+\beta_{film}\right)$$

Here, λ is the learnable residual coefficient, used to control the modulation strength. This design adaptively corrects the amplitude and bias of the positional encoding according to local geometric reliability. As a result, misleading positional cues in depth-ambiguous regions are reduced during attention matching. DM-HPE adds only lightweight 1 × 1 projections and a small MLP to the positional modulation process. It significantly reduces positional errors in regions with depth discontinuities.

### 2.3. Temporal Query Filter with Kalman-Style Temporal Refinement (TQF)

The encoder of BEVFormer updates BEV queries layer by layer through temporal self-attention (TSA) and spatial cross-attention (SCA). However, in highly dynamic and heavily occluded scenarios, cross-frame drift of BEV queries remains common. On one hand, the sampling offsets of deformable attention can increase significantly in depth-ambiguous or locally missing regions. On the other hand, the attention weights may become more dispersed, resulting in unstable aggregation outcomes. To mitigate this issue, we introduce a Temporal Query Filter (TQF) between the encoder output and decoder input. TQF treats the BEV query as the state of a dynamic system and applies a Kalman-style update to provide uncertainty-guided temporal refinement at the feature level. Its role is not to produce fully calibrated probabilistic uncertainty estimates, but to adaptively balance historical prediction and current observation during recursive temporal fusion. For compatibility with high-dimensional BEV feature representations, TQF adopts the following design choices:

- Model-free: Instead of predefining the state transition matrix F and observation matrix H, the dynamics are implicitly learned through deformable attention.
- Learnable variance: The estimation of process noise Q and observation noise R is converted into a regression task for a lightweight network, rather than using predefined statistical parameters.
- Diagonal approximation: To keep the update tractable for high-dimensional BEV queries (H × W × C), a per-element variance estimation is used, sacrificing explicit spatial-channel correlation modeling for computational feasibility within the BEVFormer pipeline.

Therefore, TQF should be understood as a data-driven, Kalman-style temporal refinement strategy based on diagonal variance approximation, rather than a fully calibrated probabilistic uncertainty model.

#### 2.3.1. State Alignment and Prediction

Let the encoder output of the t frame (unfiltered) be $z_{t}\in R^{H\times W\times C}$, and the filtered state from the previous frame be ${\hat{x}}_{t-1}\in R^{H\times W\times C}$. To eliminate the BEV grid coordinate changes caused by vehicle motion, we first align the previous frame’s state to the current frame’s coordinate system, forming the predicted state:

$${\hat{x}}_{t|t-1}\;=\;W\left({\hat{x}}_{t-1};\Delta s_{t}\right),$$

Here, W(·) represents the translation/resampling operator on the BEV grid (equivalent to common shift alignment mechanisms), and $\Delta s_{t}$ is the relative pose increment between consecutive frames. The aligned state ${\hat{x}}_{t|t-1}$ and the current frame observation $z_{t}$ are in the same BEV coordinate system, allowing subsequent innovation terms to capture the deviation between the “state after temporal propagation” and the “current observation.”

#### 2.3.2. Uncertainty Modeling via Deformable Attention Statistics

TQF constructs observation uncertainty features using internal statistics from the spatial cross-attention (SCA) module. Let the attention weights output by the SCA module be $W\in R^{B\times H\times W\times N_{head}\times N_{level}\times N_{point}}$, and the sampling offsets be $O\in R^{B\times H\times W\times N_{head}\times N_{level}\times N_{point}\times 2}$. The uncertainty statistics are extracted from two dimensions as follows:

- Attention weight dispersion: The attention weights are normalized along the head, level, and point dimensions, and their statistical features are calculated, including the mean $\mu_{w}$ and standard deviation $\sigma_{w}$. The standard deviation $\sigma_{w}$ reflects the concentration of the weight distribution: the larger $\sigma_{w}$ is, the more dispersed the attention is, and the less reliable the aggregation result becomes.
- Sampling offset stability: The sampling offsets are aggregated along the head and level dimensions to obtain the average offset and its standard deviation for each reference point across different sampling points. The magnitude of the offset $\left|\overset{-}{o}\right|$ and its spatial gradient $\nabla_{h,w}\left|\overset{-}{o}\right|$ are further calculated to characterize the spatial consistency and local continuity of the offsets. These 5-dimensional statistics depend solely on the intermediate output of the attention process and do not require additional information or geometric supervision, making them a general uncertainty measure that can be inserted into the BEV encoding pipeline.

**Figure 3:**
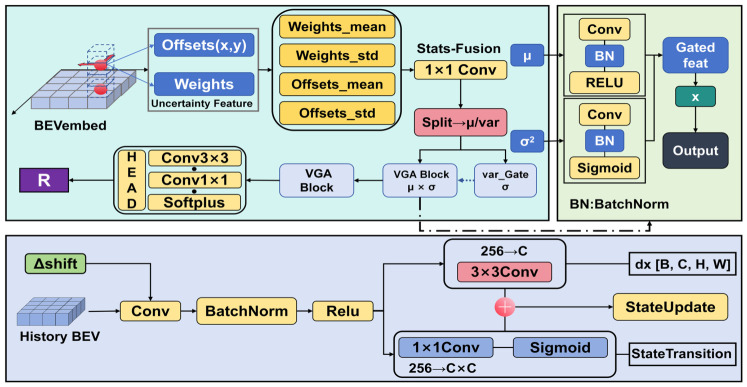
Deep learning framework for spatial uncertainty modeling based on the Variance-Gated Attention mechanism and state transition matrix modeling.

For the observation noise variance gating estimate $R_{t}^{\mathrm{obs}}$, to explicitly map observation uncertainty to observation noise covariance, we design an observation noise processing module based on Variance-Gated Attention (VGA) shown in Figure 3. Unlike traditional channel attention that generates weights using global pooling, the core idea of VGA is to directly drive the gating weight generation using the standard deviation σ of the input statistics, thus enabling a direct propagation of uncertainty from attention fluctuation to observation noise.

For process noise $Q_{t}^{\mathrm{proc}}$, this study adopts a simplified learned modeling approach: it is treated as a learnable spatial-channel shared parameter (or based on predicted residual estimation), reflecting the inherent uncertainty in the state transition process.

#### 2.3.3. Learnable Process and Observation Noise Estimate

To introduce adaptive confidence cues into the filtering update, we learn process and observation variance maps as diagonal, per-element quantities, thereby avoiding the computational cost of maintaining full covariance matrices. Specifically, observation noise $R_{t}^{\mathrm{obs}}$ represents the reliability of the current frame BEV feature aggregation and is directly mapped from the SCA module. Given the observation uncertainty features $U_{obs}$, we first separate them along the channel dimension into mean features $\mu_{u}$ and standard deviation features $\sigma_{u}$:

$$\mu_{u}={Conv}_{1\times 1}^{\left(\mu \right)}\left(U_{obs}\right)$$

$$\sigma_{u}={Conv}_{1\times 1}^{\left(\sigma \right)}\left(U_{obs}\right)$$

The gating weights of VGA are generated through the standard deviation branch:

$$g_{R}=\sigma \left({Conv}_{1\times 1}\left(BN\left(ReLU\left({Conv}_{1\times 1}\left(\sigma_{u}\right)\right)\right)\right)\right)$$

where σ(·) is the Sigmoid activation function. The physical meaning of this design is that positions with a large standard deviation $\sigma_{u}$ will produce gating weights close to 1, thereby assigning higher noise variance in the subsequent RRR estimation. After transformation, the mean features are element-wise multiplied with the gating weights:

$$f_{R}={Conv}_{1\times 1}\left(\mu_{u}\right)⊙g_{R}$$

Thus, the given observation noise $R_{t}^{obs}$ represents the reliability of the current frame BEV feature aggregation, estimated via the VGA mechanism from the SCA statistics:

$$R_{t}^{obs}={\mathrm{Softplus}(\mathrm{Conv}}_{3\times 3}\left({Conv}_{3\times 3}\left(f_{R}\right)\right))+\epsilon$$

where ϵ is a stability term. The core advantage of VGA is that it uses the standard deviation feature σ to directly generate spatial-channel adaptive gating weights, allowing the noise estimation to finely capture the differences in observation reliability across different regions and feature channels in the BEV plane. Compared to channel attention generated through global pooling (such as in SE-Net [24] or Convolutional Block Attention Module (CBAM) [25]), VGA preserves the heterogeneity of the spatial dimension, making it more suitable for handling reliability differences in near-field/far-field and occluded/visible regions in BEV perception.

This design makes the noise scale spatially adaptive. Locations with more dispersed attention or less stable sampling offsets are assigned larger variances, which lowers the update confidence in those regions. Conversely, in locations with concentrated attention and stable sampling, the variance is tightened, thereby enhancing the effective gain of temporal fusion.

Process noise $Q_{t}^{\mathrm{proc}}$ represents the uncertainty in the state transition process and is derived from the prediction–observation residual and ego-motion confidence:

$$e_{t-1}\;=\;∥{\hat{x}}_{t-1}-z_{t-1}∥$$

which reflects the historical prediction error. In practice, the magnitude $∥e_{t-1}∥$ of the previous frame innovation term is concatenated with the current ego-motion velocity, and a lightweight MLP generates $Q_{t}^{\mathrm{proc}}$:

$$Q_{t}^{\mathrm{proc}}\;=\;\mathrm{softplus}\left(g_{Q}(∥e_{t-1}∥,∥\Delta_{t}∥)\right)\;+\;\epsilon$$

This design increases the process noise when the historical prediction error is large or when ego-motion is fast. As a result, the model places more trust in the current observation. The diagonal state variance $P_{t-1}\in R^{H\times W\times C}$ is updated element-wise:

$$P_{t|t-1}\;=\;P_{t-1}\;+\;Q_{t}^{\mathrm{proc}}$$

This update treats the process noise as an additional source of uncertainty during temporal propagation, allowing the predicted variance to dynamically adjust based on scene complexity and attention stability.

**Figure 4:**
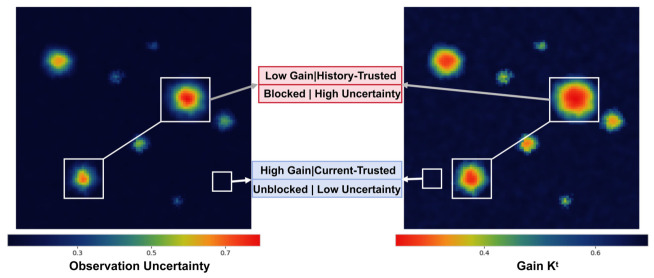
In regions of high uncertainty, K^t^ is approximately 0.6–0.7, corresponding to a weight ratio of historical prediction to current observation of about 3:7 to 4:6. This helps suppress noise while maintaining the flow of information, without excessively suppressing the current encoder output.

In summary, the learned process and observation variance maps provide complementary confidence cues for temporal fusion shown in Figure 4. The former reflects transition-related uncertainty from historical residuals and ego-motion, whereas the latter reflects observation reliability estimated from current-frame attention statistics. Both are represented as diagonal variance maps and participate in the subsequent gain calculation and state update. This design enables uncertainty-guided temporal refinement with a tractable update form, but it does not explicitly model full spatial-channel correlations or constitute a fully calibrated uncertainty estimator.

#### 2.3.4. Diagonal Covariance Update

The innovation term (the cross-frame difference after alignment) is defined as:

$$e_{t}\;=z_{t}\;-\;{\hat{x}}_{t|t-1}.$$

Based on the diagonal form of the predicted variance $P_{t|t-1}\;$ and the observation noise $R_{t}^{\mathrm{obs}}$, the gain and state update are computed as follows:

$$K_{t}\;=\;\frac{P_{t|t-1}}{P_{t|t-1}+R_{t}^{\mathrm{obs}}},\;{\hat{x}}_{t}\;=\;{\hat{x}}_{t|t-1}\;+\;K_{t}⊙e_{t},$$

where the operations are performed element-wise, and ⊙ denotes element-wise multiplication. The Kalman-style gain $K_{t}$ adapts both spatially and across channels, enabling differential control of update strength across different regions of the BEV grid. The variance is synchronized and updated in a diagonal form:

$$P_{t}\;=\;\left(1\;-{\;K}_{t}\right)⊙P_{t|t-1}$$

This allows the propagated uncertainty to be passed on to the next frame. Initialization is set as ${\hat{x}}_{0}\;=\;z_{0}$ and $P_{0}\;=\;\lambda I$, where λ is a constant scalar, providing a stable initial uncertainty scale and preventing numerical oscillations during early training stages.

#### 2.3.5. Uncertainty-Aware Training Objective

To form a closed loop among variance prediction, gain computation, and state updating, we apply a Gaussian negative log-likelihood constraint to the innovation term. This objective encourages the learned diagonal variance to respond adaptively to the innovation magnitude, but it is not intended as a dedicated probabilistic calibration procedure. The diagonal innovation variance $S_{t}\;=\;P_{t|t-1}^{-}+R_{t}^{\mathrm{obs}}$ is used, and the loss is defined as:

$$L_{\mathrm{TQF}}\;=\;\frac{1}{2}\left(\log\left(S_{t}\;+\;\epsilon \right)\;+\;\frac{e_{t}^{2}}{S_{t}+\epsilon }\right)$$

where the mean is taken over the spatial dimensions (H, W, C). This loss function simultaneously constrains both the innovation magnitude and the variance scale: when the alignment error is large, the model is required to increase St to reduce overly confident updates; when the alignment error is small, the model tends to tighten St to improve the effectiveness of the fusion. With this objective, TQF provides a more stable temporal refinement signal for the decoder. It is not intended to produce a strictly calibrated posterior uncertainty estimate.

### 2.4. Query Masking for BEV Regularization

**Figure 5:**
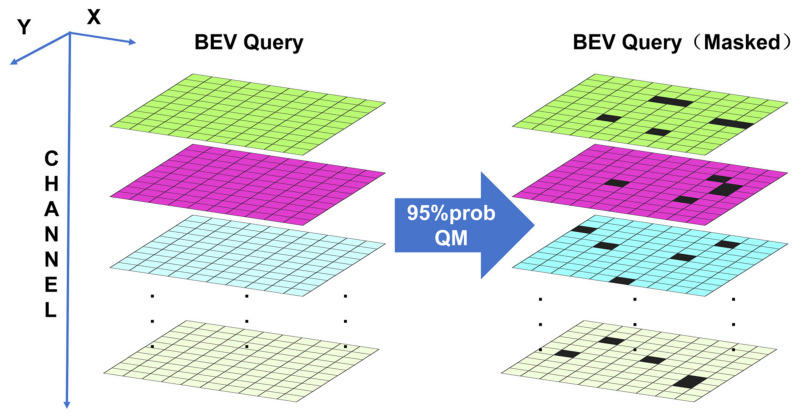
The random Query Mask applied to BEV features, with channel-wise and grid-wise masking.

Although DM-HPE and TQF enhance geometric representation and temporal consistency, BEV queries may still over-rely on local visible cues during training, which can reduce robustness under sudden occlusion, local view loss, or image degradation. To address this issue, we introduce Query Mask (QM), a structured training-time regularization strategy that operates directly in the BEV query space. As shown in Figure 5, unlike image-domain occlusion augmentation, QM applies structured dropout to the BEV query along the feature propagation path, encouraging the encoder to maintain stable updates under partial query corruption. QM is activated only during training and bypassed during inference.

#### 2.4.1. Spatial Mask Construction in BEV Space

QM adopts a spatially continuous masking pattern to simulate the structural nature of real-world occlusions. Specifically, on a low-resolution BEV grid (e.g., H/4 × W/4), a coarse mask is sampled with a spatial retention rate p_s_, and then upsampled to H × W using nearest-neighbor interpolation, ensuring that the masked regions exhibit a block-connected distribution. Afterward, morphological dilation is applied to the regions where mask = 0, adjusting the connectivity and coverage of the occlusion blocks. This process explicitly controls the shape and intensity of spatial missingness with a small number of parameters, making it easier for ablation studies and reproducibility.

#### 2.4.2. Spatial-Channel Joint Masking Strategy

Simply performing grid-level masking is equivalent to “simultaneously weakening all channels in a grid,” resulting in a relatively uniform missing pattern. To enhance the granularity and coverage of the missing pattern, QM introduces channel-level masking on top of the spatial mask. This allows the mask to not only determine “which BEV grids are weakened,” but also “which channels within the same grid are weakened.” The combined mask has the same shape as the BEV query: [B, H × W, C].

Channel masking adopts a fixed-ratio strategy: Let the channel retention rate be p_c_. For each BEV grid location, approximately p_c_ C channels are retained, with the remaining channels set to zero. In implementation, for each (h,w), a fixed-size subset is sampled from the set of channels to form the retained subset, ensuring a stable proportion of channel deactivation at each grid location (avoiding fluctuations in the number of retained channels caused by element-wise Bernoulli sampling). Additionally, the retained subset changes across different positions and iterations to prevent pattern fixation. For example, with C = 256 and p_c_ = 0.9, each grid location will have approximately 25 channels set to zero, encouraging the network to leverage the redundant representation in the remaining channels to complete inference and updates.

#### 2.4.3. Integration into Encoder and Computational Overhead

The QM is applied between TSA (Temporal Self-Attention) and SCA (Spatial Cross-Attention): specifically, after TSA aggregates historical information and before SCA fuses multi-view features, the BEV query is masked, ensuring that subsequent updates rely more fully on cross-view context. The core operation is element-wise multiplication of the BEV query with the mask:

$$Q^{\prime }=Q⊙M.$$

The mask generation consists of low-resolution sampling, nearest-neighbor upsampling, and dilation, resulting in a relatively negligible computational overhead compared to the main encoder. The module does not introduce learnable parameters, making it easily integrable into any BEV architecture without changing the network structure. Thus, QM is enabled during training and disabled during inference.

## 3. Results

To validate the performance of the proposed algorithm, experiments were conducted on the nuScenes dataset. We report the nuScenes detection score (NDS), mean average precision (mAP), and the standard true-positive error metrics, including mean translation error (mATE), mean scale error (mASE), mean orientation error (mAOE), mean velocity error (mAVE), and mean attribute error (mAAE), and the AdamW optimizer was used. The initial learning rate was set to 0.0002 and decayed to 0.00001 via cosine annealing. Due to memory constraints, training was conducted for 24 epochs on two RTX 4090 GPUs (24 GB each) with a batch size of 1. One GPU was dedicated to training the Base model and its variants, while the other handled the Tiny model configurations. The checkpoint with the best validation performance was selected as the final model. The average inference time on the validation set, excluding data loading overhead, was measured with a batch size of 1 on a single RTX 4090 GPU, following the MMDetection3D evaluation protocol.

All ablation studies used the same training configuration (AdamW, 24 epochs, batch size 1) to ensure a fair comparison. The reproduced baseline in this study is consistent with the original BEVFormer-base (ResNet-101) model from the related paper. This section presents the experimental results of the proposed TriQuery-BEV model in the 3D perception tasks of autonomous driving. The experiments include comparisons with representative BEV-3D perception models and ablation studies of the proposed modules. Overall inference time is measured under the MMDetection3D evaluation protocol. The ablation experiments verified the effectiveness of the Query Mask (QM), Temporal Self-Attention (TSA), and Temporal Query Filter (TQF) modules, particularly their performance improvements in handling issues such as local occlusion, geometric uncertainty, and temporal drift. The results demonstrate the robustness of TriQuery-BEV in complex autonomous driving scenarios.

### 3.1. Comparison with State-of-the-Art Methods

This study compares the proposed TriQuery-BEV with the baseline BEVFormer and other state-of-the-art (SOTA) BEV detectors listed in Table 1. The performance of different model configurations was evaluated, specifically applying TriQuery-BEV to the baseline tiny and base variants. Comparative experiments were conducted with mainstream open-source SOTA models.

The comparative experiments show that TriQuery-BEV consistently achieves performance gains over BEVFormer across both model scales, with improvements not only in detection scores but also in the systematic reduction in multiple error metrics. This suggests that query updates lead to synergistic benefits in spatial geometric constraints, cross-frame consistency, and occlusion robustness. For instance, with the R101 backbone configuration, TriQuery-BEV-base achieves NDS and mAP scores of 54.8 and 44.3, respectively, which represent a 6.0% and 6.5% improvement over BEVFormer-base (51.7 and 41.6). Simultaneously, mATE decreases by 6.5%, mASE decreases by 6.6%, mAOE decreases by 8.3%, mAVE decreases by 15.0%, and mAAE decreases by 12.6%, indicating more stable predictions for object localization, scale, orientation, speed, and attributes. The same trend is maintained under the lightweight R50 configuration, with TriQuery-BEV-tiny showing a 5.4% increase in NDS (35.5→37.4) and a 6.4% increase in mAP (25.1→26.7), while mATE and mAOE continue to decline, showing that the method’s gains do not rely on model capacity stacking and still provide reliable improvements even under low-computation configurations.

From an error decomposition perspective, the advantages of TriQuery-BEV come not from a single component but from the synchronized convergence of geometric and temporal errors. The decrease in mATE indicates more accurate object center regression, reflecting more stable cross-view correspondences and reduced spatial bias due to depth ambiguity. The reduction in mAOE corresponds to more consistent heading estimation, indicating that the direction-sensitive representation of the BEV query is less prone to local occlusion or viewpoint changes during cross-frame updates. The improvement in mASE indicates more consistent scale regression, which reflects better fidelity of the BEV spatial representation to the object’s shape. The significant decrease in mAVE reveals that temporal updates are smoother for dynamic objects, effectively suppressing drift accumulation. The reduction in mAAE suggests that attribute prediction is less sensitive to temporal noise, enhancing the consistency of multi-frame decision-making.

In summary, these results align with the design goal of “explicit geometric modulation at the query level and the introduction of uncertainty constraints for temporal updates.” Compared to methods like DETR3D, which directly decode in the image domain, TriQuery-BEV shows clear advantages in both main metrics and key error terms. This validates that when BEV representations unify spatial structures into the vehicle coordinate system, they are better able to implicitly incorporate extrinsic geometric constraints into attention aggregation, thereby improving regression stability for long-range targets and long-tail poses. When compared to solutions like PETR, which emphasize 3D coordinate encoding, the results of TriQuery-BEV show that relying solely on coordinate priors still struggles to cover the sources of temporal uncertainty in complex dynamic scenes. Explicit uncertainty modeling in the query update process appears more effective in achieving cross-frame consistency convergence. In the comparison with BEVDet4D, the two models have differences in their emphasis on error terms, but TriQuery-BEV stands out in detection accuracy improvement and the overall reduction in multiple geometric errors, demonstrating the effectiveness and scalability of query-level regularization and temporal consistency enhancement in complex occlusion and dynamic traffic scenarios.

### 3.2. Ablation Studies

#### 3.2.1. Analysis of Positional Encoding Strategies

In this section, we conduct an ablation study on positional encoding using the nuScenes validation set. All experiments are conducted with the same training configuration and a fixed ResNet-101 backbone. Four configurations are compared:

- BEVFormer original learnable positional encoding: This serves as the baseline with purely learnable positional encodings.
- Fixed Random Fourier Features: This configuration uses only fixed Random Fourier Features (RFF) as the positional encoding, testing the replaceability of pure geometric priors.
- DM-HPE with Sine Priors: In this configuration, the sine position priors are added to the learnable encoding, and channel-level modulation is performed using depth reliability gating.
- DM-HPE with Random Fourier Priors: This setup is similar to the third configuration but replaces the sine prior with the Random Fourier Position Encoding used in the proposed model. This comparison aims to assess the impact of different geometric prior forms.

These configurations help evaluate the contributions of different positional encoding strategies to the overall model performance in terms of positional accuracy, robustness, and consistency.

The experimental results presented in Table 2 demonstrate that relying solely on fixed geometric encodings leads to significant performance degradation. Combining fixed geometric priors with learnable encodings and modulating them via depth reliability gating yields stable improvements in detection metrics, indicating that positional encoding directly influences the geometric alignment and cross-frame consistency of BEV queries.

Using BEVFormer’s learnable positional encoding as the baseline (NDS/mAP: 35.5/25.1 for Tiny, 51.7/41.6 for Base), replacing it with fixed RFF encoding causes substantial performance drops: NDS decreases by 4.5% and 8.3%, while mAP decreases by 0.8% and 5.8% for Tiny and Base, respectively. This degradation indicates that fixed encodings lack adaptability to task distributions and fail to compensate for systematic errors and occlusion-induced information loss during multi-view-to-BEV association. In depth-ambiguous regions, unstable spatial correspondences weaken both query separability and temporal transferability.

With the introduction of DM-HPE, performance is restored and exceeds baseline levels across both model scales. DM-HPE with sinusoidal prior improves NDS/mAP by 2.0%/1.2% (Tiny) and 1.5%/0.7% (Base), while RFF prior achieves gains of 3.1%/2.0% (Tiny) and 2.3%/1.7% (Base). These results suggest that the hybrid strategy benefits from the combination of learnable residual adaptation, fixed geometric priors, and reliability-guided modulation. The observed gains are consistent with the intended role of depth-reliability gating in reducing the influence of unstable positional cues in depth-uncertain regions.

Notably, RFF prior consistently outperforms sinusoidal prior in mAP improvement, particularly for the Base model, suggesting that richer spectral bases better capture fine-grained spatial variations in BEV grids when model capacity is sufficient. Conversely, hybrid encodings without depth gating modulation (RFF + learnable PE only) exhibit smaller gains and fail to surpass full DM-HPE performance, especially in the Base configuration. This suggests that removing depth gating weakens the effectiveness of the hybrid positional modulation, which may limit robustness under occlusion and geometric ambiguity. In summary, depth gating modulation is crucial for enhancing BEV query stability and robustness.

These results suggest that the learned modulation mechanism in DM-HPE contributes to the observed gains beyond simply combining fixed and learnable positional priors. However, the present study does not provide a standalone evaluation of depth-gate quality or explicit depth supervision, and the current evidence should therefore be interpreted as task-level support for geometry-aware modulation rather than a direct validation of depth estimation accuracy.

#### 3.2.2. Analysis of Temporal Fusion Mechanisms

To validate the design choices of the observation uncertainty estimation head (R-Head) in TQF, an ablation study was conducted on the attention mechanism within R-Head. R-Head extracts observation noise through attention statistics, and this experiment compares three implementations: Variance-Gated Attention (VGA), Shuffle Attention [27] (SA), and the original CBAM. Due to computational resource constraints, the Tiny configuration only verifies the effectiveness of VGA, while the Base configuration further compares the three attention mechanisms to identify the optimal design for R-Head.

Through these experiments, this study analyzes the role of the Query Filter and different attention mechanisms in improving the model’s robustness and temporal consistency. The results will help determine which attention mechanism is most effective in addressing the uncertainty in observation noise and contributing to enhanced model performance in dynamic and occluded environments.

The ablation study results shown in Table 3 indicate that the TQF module improves the performance of the BEVFormer model across several metrics. For the BEVFormer-tiny model, after adding TQF (VGA), NDS improves by 1.9%, mAP improves by 2.3%, and mAVE decreases by 1.0%, indicating that TQF enhances the model’s temporal consistency and robustness. For the BEVFormer-base model, introducing TQF (VGA) results in a 2.6% increase in NDS, a 3.8% increase in mAP, and a 5.7% decrease in mAVE, highlighting the significant improvement in dynamic target speed estimation through uncertainty modeling. Additionally, mATE decreases by 3.5%, showing that temporal filtering effectively suppresses position drift. These results also provide task-level evidence that the proposed diagonal-variance TQF improves temporal consistency and robustness in the intended detection setting, although they do not constitute a full validation of probabilistic calibration or richer covariance modeling.

The comparison of the three attention mechanisms reveals key factors in the design of R-Head. VGA achieves the best overall performance, followed by SA, while the original CBAM performs slightly worse. VGA provides a relative gain of 0.9% in NDS over CBAM, suggesting that a variance-gated design is more suitable for uncertainty estimation. Although SA slightly outperforms VGA in mASE, its weaker performance in mAVE and mAAE suggests that uniform channel mixing may disrupt the spatial distribution of uncertainty statistics.

Overall, VGA better matches the modeling requirements of observation noise through its dual-branch channel-space design and variance-gating mechanism. It shows particularly strong performance in dynamic target speed estimation (mAVE) and attribute prediction (mAAE). The addition of TQF shows notable improvements in error decomposition, with decreases in mATE and mAOE, indicating that temporal filtering successfully reduces target localization and heading errors, further validating the enhancement of temporal consistency.

In summary, TQF significantly improves the temporal consistency and robustness of the BEVFormer model by explicitly modeling temporal uncertainty, especially enhancing performance in dynamic environments. The comparison between VGA, SA, and CBAM further underscores the necessity of designing attention mechanisms for uncertainty estimation tasks. VGA’s variance-gated structure provides superior observation noise modeling capabilities in scenarios with occlusion and high uncertainty.

#### 3.2.3. Analysis of Query Masking Strategies

To evaluate the effectiveness of Query Mask (QM), several experiments were designed. First, for the BEVFormer-base model, we compared the original model with a version incorporating random Query Mask (QM (random)) to assess the impact of Query Mask on performance improvement. Due to computational resource and time limitations, the models with different masking strategies were based on the BEVFormer-tiny model. We evaluated different retention probabilities (0.89, 0.92, and 0.95) and compared them with the random probability Query Mask used in TriQuery-BEV, testing the effect of different masking strategies in dynamic scenes. These experiments evaluate the impact of Query Mask across different model scales, especially its effectiveness in improving temporal consistency and occlusion robustness.

The ablation experiment analysis of Query Mask (QM) presented in Table 4 shows that the impact of the QM strategy on model performance is configuration-dependent. In the BEVFormer-base model, random Query Mask (QM (random)) improves NDS by 0.9% and mAP by 0.9%, indicating that QM effectively enhances temporal consistency and occlusion robustness in larger models. For the BEVFormer-tiny model, with fixed mask probabilities (0.89/0.92/0.95), the NDS values are 37.1, 37.3, and 37.3, and the mAP values are 26.5, 26.6, and 26.6, showing an upward trend as the retention rate increases. However, the NDS at 0.89 is slightly lower than the baseline (37.1 vs. 37.2), which may be due to excessive masking leading to information loss. The random Query Mask (QM (random)) achieves NDS of 37.4 and mAP of 26.8, outperforming all fixed probability configurations and demonstrating the advantage of a randomized masking strategy in preventing pattern fixation.

The gain from QM is more significant in the Base model (NDS +0.9% vs. Tiny +0.5%), reflecting a larger benefit from the regularization strategy in models with higher capacity. The fixed probability masks show a saturation of gains in the range of 0.89–0.95 (with NDS increasing from 37.1 to 37.3, a gain of only 0.2), suggesting that a single fixed mask strength is insufficient to adapt to diverse occlusion scenarios.

These results suggest that a single fixed mask strength is insufficient to cover diverse occlusion patterns during training. By contrast, random masking introduces more varied structured missingness and leads to better robustness under partial information loss. Since QM is used only during training and is bypassed during inference, its benefit should be understood as improved representation robustness learned during training rather than as an explicit test-time completion mechanism. The current evidence therefore supports QM as a training-time regularizer for partial-query robustness, but does not constitute a direct validation of “local missing → global completion” behavior.

### 3.3. Robustness Under Adverse Conditions and Comparison with an EMA-Style Temporal Baseline

To further evaluate the proposed method under adverse conditions and to isolate whether the temporal gain comes from the Kalman-style structure rather than generic temporal denoising, we conduct an additional robustness experiment on a representative subset of the RoboBEV/nuScenes-C benchmark [28]. RoboBEV is a corruption-oriented benchmark for camera-based BEV perception built upon nuScenes, and its corruption suite covers realistic adverse conditions including weather degradation, image distortion, and camera failure. In this study, we use six representative corruptions—Fog, Low Light (Dark), Motion Blur, Frame Lost, Camera Crash, and Snow—together with the clean setting.

Due to the added experimental cost, this supplementary robustness study is conducted on the BEVFormer-small setting, and all compared methods follow the same training and evaluation protocol. To distinguish generic temporal smoothing from the adaptive Kalman-style refinement introduced by TQF, we compare three settings under the same backbone and training protocol: (1) the original baseline, (2) exponential moving average (EMA)-style temporal smoothing baseline, and (3) the full TriQuery-BEV. The EMA baseline performs fixed-coefficient recursive smoothing on the BEV query:

$$Q_{t}^{EMA}=\alpha Q_{t}+\left(1-\alpha \right)Q_{t-1}^{EMA}$$

where α = 0.8 is fixed for all scenarios. Unlike TQF, this baseline does not estimate uncertainty and does not adapt the fusion strength according to the reliability of the current observation.

The experimental results of this comparative study are summarized in Table 5. For the comprehensive results including all detailed true-positive error metrics, please refer to Appendix A.

As shown in Table 5, the EMA baseline provides only marginal gains under relatively mild conditions. For example, NDS increases from 0.479 to 0.481 on the clean setting and from 0.358 to 0.361 under Fog. However, under more severe corruptions such as Motion Blur, Frame Lost, Camera Crash, and Snow, EMA becomes unstable and underperforms the original baseline. In contrast, TriQuery-BEV consistently achieves the best results across all reported conditions. Relative to EMA, TriQuery-BEV improves NDS by +0.018 on Clean, +0.027 on Fog, +0.024 on Low Light, +0.029 on Motion Blur, +0.034 on Frame Lost, +0.032 on Camera Crash, and +0.030 on Snow. Averaged over the six corrupted conditions, this corresponds to an improvement of approximately +0.029 NDS over EMA. Similar improvements are also observed in mAP and mAVE, with particularly clear reductions in mAVE under severe degradations such as Fog, Frame Lost, and Snow.

These results support the intended role of TQF. EMA applies the same fixed fusion coefficient to all frames regardless of corruption severity. By contrast, TQF uses learned diagonal variance together with the Kalman gain to adapt the update strength at the query level. When the current observation becomes unreliable, TQF can reduce the influence of the corrupted observation and rely more on the propagated state, rather than blindly averaging degraded information. This difference becomes more important under severe corruptions, which is consistent with the larger performance gap observed in Motion Blur, Frame Lost, Camera Crash, and Snow.

## 4. Discussion

TriQuery-BEV is a modular BEV perception framework designed to improve 3D perception in autonomous driving, particularly in dynamic scenes with occlusion and recursive temporal fusion. By integrating Query Mask (QM), Depth-Modulated Hybrid Positional Encoding (DM-HPE), and Temporal Query Filter (TQF), the proposed method improves detection accuracy and temporal consistency over the BEVFormer baseline. Experimental results on nuScenes show gains in NDS and mAP, together with reductions in key error metrics such as mATE, mAOE, and mAVE. These results suggest improved robustness in localization, orientation estimation, and temporal refinement under challenging visual conditions.

**Figure 6:**
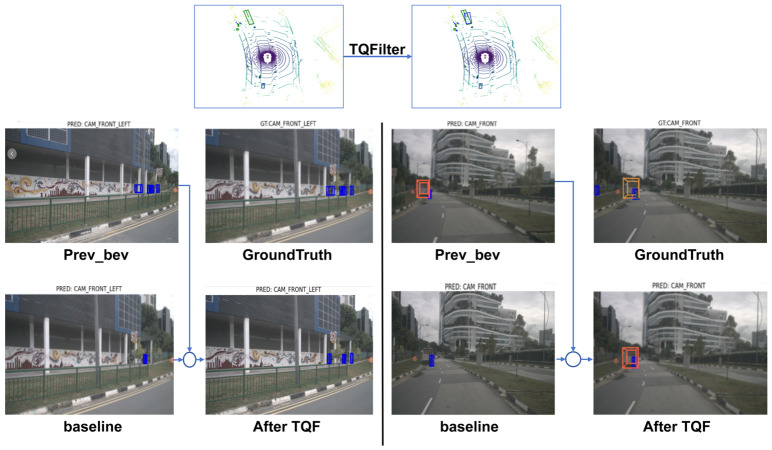
The comparison of the 3D perception results of BEV perception and surround-view cameras, including the front-facing and left front-facing cameras, after processing by the baseline and TQF, along with an overview of the process. The image itself was not involved in the processing. This type of visualization is used for illustrative purposes after filtering. It can be observed that when the previous frame is detected accurately, the visualization of the next frame’s detection results is significantly better than the baseline output. In the visualization, blue bounding boxes denote pedestrian detection, orange bounding boxes represent ground-truth vehicle results, and red bounding boxes indicate predicted vehicle results. In the BEV visualization, green bounding boxes represent ground-truth boxes, while blue bounding boxes denote detection results.

The qualitative visualization results are presented in Figure 6. Nevertheless, the present study still has several limitations. The newly added robustness study provides additional task-level evidence under adverse visual conditions, but the evaluation remains limited to nuScenes and a representative RoboBEV/nuScenes-C subset. Therefore, the generalization ability of the framework under more diverse real-world environments, longer temporal horizons, and denser traffic interactions remains to be further validated. More broadly, recent studies in intelligent transportation systems have shown that system reliability under external perturbations depends strongly on robustness and recovery characteristics [29].

Although TQF improves temporal consistency in our task-level evaluations, its uncertainty modeling remains approximate. Specifically, TQF adopts a diagonal variance formulation for computational feasibility and does not model full spatial-channel correlations. Moreover, the current study does not provide a dedicated calibration analysis or comparisons with richer covariance parameterizations such as full-covariance or low-rank alternatives. Therefore, the learned variance should be interpreted as a feature-level confidence cue for query-level temporal fusion rather than a fully calibrated probabilistic uncertainty estimate.

DM-HPE also has important limitations. Although it uses a learned depth-reliability gate for positional modulation, the current study does not include explicit depth supervision, a standalone depth-accuracy evaluation, a region-specific analysis in highly ambiguous areas such as overpasses, or a separate ablation on the depth-supervision component. Thus, the present evidence supports the task-level usefulness of the modulation mechanism, but not a direct claim of improved depth estimation accuracy.

In addition, QM functions as a structured training-time regularizer and is bypassed during inference. Its contribution to robustness is therefore indirect, through improved representation learning under partial query corruption during training, rather than through an explicit test-time completion mechanism. Although the current study compares several fixed masking ratios with the random masking strategy adopted in TriQuery-BEV, it does not include a dedicated heavy-occlusion benchmark, region-level visualization, or quantitative analysis of completion behavior.

Finally, the current study reports only overall inference time on a single RTX 4090 GPU and does not provide a detailed latency breakdown for each module. In addition, deployment feasibility on embedded platforms has not been directly evaluated. These aspects remain important directions for future work. Future research will therefore focus on broader robustness validation, richer uncertainty parameterizations, more detailed analysis of geometry-aware modulation, and deployment-oriented optimization.

## Figures and Tables

**Table 1:** The comparative experiments of different mainstream BEV perception models.

Model	backbone	NDS	mAP	mATE	mASE	mAOE	mAVE	mAAE
BEVFormer-tiny	r50	35.5	25.1	0.898	0.293	0.651	0.657	0.216
TriQuery-BEV-tiny	r50	37.4	26.7	0.872	0.287	0.614	0.609	0.215
BEVFormer-base	r101	51.7	41.6	0.673	0.274	0.372	0.394	0.198
TriQuery-BEV-base	r101	54.8	**44.3**	0.629	**0.256**	**0.341**	**0.335**	0.173
DETR3D	r101	42.5	34.6	0.773	0.268	0.383	0.842	0.216
PETR	vovnet	45.5	40.3	0.736	0.271	0.432	0.825	0.204
BEVdet4D [26]	r101	**55.3**	42.9	**0.619**	0.271	0.351	0.386	**0.155**

Bold values indicate experimental results that outperform other models.

**Table 2:** Ablation Study on Deep Gated Hybrid Positional Encoding.

Ablation of Methods	Tiny		Base	
	NDS	mAP	NDS	mAP
BEVFormer(LPE)	35.5	25.1	51.7	41.6
BEVFormer with DM-HPE(Sinusoidal)	36.2	25.4	52.5	41.9
BEVFormer with DM-HPE(RFF)	36.6	25.7	52.9	42.3
BEVFormer with RFF	33.9	24.9	47.4	39.2
BEVFormer with Hybrid PE (Unmodulated)	35.6	25.0	51.9	41.6

**Table 3:** Ablation study on Query Filter with different attention mechanisms.

Ablation of Methods	NDS	mAP	mATE	mASE	mAOE	mAVE	mAAE
BEVFormer-tiny *	36.5	25.7	0.879	0.288	0.632	0.623	0.216
BEVFormer-tiny * with Query-Filter (VGA)	37.2	26.3	0.871	0.288	0.611	0.617	0.215
BEVFormer-base *	52.9	42.3	0.661	0.263	0.355	0.369	0.194
BEVFormer-base * with Query-Filter (VGA)	**54.3**	**43.9**	**0.638**	0.261	**0.344**	**0.348**	**0.184**
BEVFormer-base * with Query-Filter (SA)	54.0	**43.9**	0.640	**0.259**	0.351	0.357	0.189
BEVFormer-base * with Query-Filter (CBAM)	53.8	43.5	0.642	0.263	0.351	0.360	0.189

* The compared Query Filter variants are evaluated on a framework already equipped with DM-HPE modules. Bold values indicate experimental results that outperform other models.

**Table 4:** Ablation Study of Query Mask (QM) Strategies.

Method	NDS	mAP
BEVFormer-base *	54.3	43.9
BEVFormer-base * + QM (random)	54.8	44.3
BEVFormer-tiny *	37.2	26.3
BEVFormer-tiny * + QM (89%)	37.1	26.5
BEVFormer-tiny * + QM (92%)	37.3	26.6
BEVFormer-tiny * + QM (95%)	37.3	26.6
BEVFormer-tiny * + QM (random)	37.4	26.8

* denotes the model with improvements to the Query Filter and DM-HPE based on the baseline.

**Table 5:** Robustness comparison on a representative RoboBEV/nuScenes-C subset and the clean setting.

Condition	Baseline NDS	EMA NDS	TQ-BEV* NDS	Baseline mAP	EMA mAP	TQ-BEV* mAP	Baseline mAVE	EMA mAVE	TQ-BEV* mAVE
Clean	0.479	0.481	0.499	0.370	0.372	0.388	0.436	0.418	0.371
Fog	0.358	0.361	0.388	0.249	0.250	0.277	0.830	0.814	0.731
Low Light	0.241	0.244	0.268	0.119	0.121	0.142	1.039	1.015	0.938
Motion Blur	0.257	0.254	0.283	0.134	0.132	0.156	0.963	0.944	0.870
Frame Lost	0.246	0.243	0.277	0.093	0.092	0.120	0.915	0.889	0.794
Cam Crash	0.277	0.274	0.306	0.113	0.111	0.137	0.838	0.816	0.740
Snow	0.181	0.179	0.209	0.064	0.062	0.084	1.100	1.075	0.977

TQ-BEV* denotes TriQuery-BEV.

## Data Availability

The data can be found at https://www.nuscenes.org/, accessed on 20 September 2025.

## Appendix A

This Table A1 reports the full results of the supplementary robustness study conducted on a representative RoboBEV/nuScenes-C subset. The evaluation includes six adverse conditions—Fog, Low Light, Motion Blur, Frame Lost, Camera Crash, and Snow—together with the clean setting. Compared with Table 5 in the main text, this table provides the complete set of reported metrics, including all true-positive error terms.

**Table A1:** Full results of the supplementary robustness study on the RoboBEV/nuScenes-C subset, including six adverse conditions and the clean setting.

Corruption	Model	NDS	mAP	mATE	mASE	mAOE	mAVE	mAAE
Clean	Baseline	0.479	0.370	0.721	0.279	0.407	0.436	0.220
	+EMA Smoothing	0.481	0.372	0.724	0.279	0.405	0.418	0.219
	TriQuery-BEV	0.499	0.388	0.697	0.267	0.371	0.371	0.208
Fog	Baseline	0.358	0.249	0.813	0.286	0.506	0.830	0.225
	+EMA Smoothing	0.361	0.250	0.817	0.286	0.508	0.814	0.224
	TriQuery-BEV	0.388	0.277	0.777	0.274	0.460	0.731	0.215
Low Light	Baseline	0.241	0.119	0.884	0.360	0.647	1.039	0.332
	+EMA Smoothing	0.244	0.121	0.886	0.361	0.645	1.015	0.332
	TriQuery-BEV	0.268	0.142	0.856	0.344	0.597	0.938	0.316
Motion Blur	Baseline	0.257	0.134	0.900	0.326	0.677	0.963	0.261
	+EMA Smoothing	0.254	0.132	0.908	0.329	0.682	0.944	0.263
	TriQuery-BEV	0.283	0.156	0.871	0.311	0.626	0.870	0.250
Frame Lost	Baseline	0.246	0.093	0.896	0.341	0.574	0.915	0.280
	+EMA Smoothing	0.243	0.092	0.910	0.344	0.578	0.889	0.283
	TriQuery-BEV	0.277	0.120	0.858	0.324	0.522	0.794	0.265
Cam Crash	Baseline	0.277	0.113	0.863	0.310	0.540	0.838	0.245
	+EMA Smoothing	0.274	0.111	0.875	0.312	0.544	0.816	0.247
	TriQuery-BEV	0.306	0.137	0.838	0.300	0.502	0.740	0.236
Snow	Baseline	0.181	0.064	0.963	0.386	0.774	1.100	0.386
	+EMA Smoothing	0.179	0.062	0.972	0.388	0.781	1.075	0.389
	TriQuery-BEV	0.209	0.084	0.919	0.369	0.717	0.977	0.366
